# Structural Characterization and In-Vitro Antioxidant and Immunomodulatory Activities of Polysaccharide Fractions Isolated from *Artemisia annua* L.

**DOI:** 10.3390/molecules27113643

**Published:** 2022-06-06

**Authors:** Lin Zhang, Narsimha Reddy, Cheang Soo Khoo, Sundar Rao Koyyalamudi

**Affiliations:** 1 Beijing Hospital of Traditional Chinese Medicine, Capital Medical University, Beijing 100010, China; yyszl@wjw.beijing.gov.cn; 2 Beijing Institute of Chinese Medicine, Beijing 100010, China; 3 School of Science, Parramatta Campus, Western Sydney University, Locked Bag 1797, Penrith, NSW 2751, Australia; 4 Wentworth Institute, 302-306 Elizabeth Street, Surry Hills, NSW 2010, Australia; khoo2031@gmail.com; 5 Institute of Endocrinology and Diabetes, The Children’s Hospital at Westmead, Sydney, NSW 2145, Australia; sundar.koyyalamudi@health.nsw.gov.au; 6 Discipline of Paediatrics and Child Health, The Children’s Hospital at Westmead, The University of Sydney, Sydney, NSW 2145, Australia

**Keywords:** *Arimisia annua* L., polysaccharides, immunomodulatory, FTIR, antioxidant

## Abstract

*Arimisia annua* L. is an important anticancer herb used in traditional Chinese medicine. The molecular basis underpinning the anticancer activity is complex and not fully understood, but the herbal polysaccharides, broadly recognised as having immunomodulatory, antioxidant and anticancer activities, are potential key active agents. To examine the functions of polysaccharides from A. annua, their immunomodulatory and antioxidant potentials were evaluated, as well as their structural characterization. The water-soluble polysaccharides (AAPs) were fractionated using size-exclusion chromatography to obtain three dominant fractions, AAP-1, AAP-2 and AAP-3, having molecular masses centered around 1684, 455 and 5.8kDa, respectively. The antioxidant potentials of the isolated polysaccharides were evaluated by measuring radical scavenging activities against DPPH^●^ (2,2-diphenyl-1-picrylhydrazyl radical), ABTS^●+^ (2,2′-azino-bis (3-ethylbenzothiazoline-6-sulphonic acid radical ion), and the OH^●^ (hydroxyl radical). AAP-1 displayed high antioxidant activities against these radicals, which were 68%, 73% and 78%, respectively. AAP-2 displayed lower scavenging activities than the other two fractions. Immunostimulatory activities of AAPs were measured using mouse macrophages. The three polysaccharide fractions displayed significant antioxidant activities and stimulated the production of tumor necrosis factor-α (TNF-α) and interleukin-6 (IL-6). AAP-1 showed significant immunostimulatory activity (16-fold increase in the production of IL-6 compared to the control and 13-fold increase in the production of TNF-α) with low toxicity (>60% cell viability at 125 μg/mL concentration). Preliminary structural characterization of the AAPs was carried out using gas chromatography (GC) and FTIR techniques. The results indicate that AAP-1 and AAP-2 are pyranose-containing polysaccharides with β-linkages, and AAP-3 is a β-fructofuranoside. The results suggest that these polysaccharides are potential candidates for immunotherapy and cancer treatment.

## 1. Introduction

*Artemisia* L. (Asteraceae) is one of the largest and most diverse genus of plants, consisting of more than 500 species and is mainly found in Asia, Europe and North America [1]. Many of these plants are widely used for medicinal purposes [1,2], such as treatment of cancer, malaria, hepatitis, inflammation and infections caused by fungi, bacteria and viruses [3].

Traditionally, *Artemisia annua* (Asteraceae) is an alternative source of nutrients for humans and livestock and is found in the northern parts of China [3]. The stems were also traditionally used in Chinese medicine for preventing malaria and enhancing immunity in patients [4,5,6,7,8,9,10,11]. *A. annua* also displayed anticancer activities against various *tumors in vitro* and in vivo, as well as exhibiting significant synergistic effects with several clinical anti-tumor medicines [6,12]. Several bioactive compounds have been isolated from *A. annua*, which include sesquiterpenoids, flavonoids, coumarins, triterpenoids, steroids, phenolics, purines and lipids [7,13,14,15,16,17,18]. The plant gained even greater attention after Professor Youyou Tu won the Nobel Prize in 2015 for discovering the antimalarial sesquiterpenoid, artemisinin from *A. annua* [4,5].

Natural polysaccharides are ideal candidates for developing novel anticancer agents due to their biological activities [19,20,21,22,23,24,25]. However, limited scientific literature exists on polysaccharides isolated from *Artemisia* [26,27]. Polysaccharides isolated from *A. apiacea* displayed significant immunomodulatory and anticancer activity [28], polysaccharide (ASKP-1) from *A. sphaerocephala* exhibited immune enhancing capacity [29], and an inulin-type fructan from *A. japonica* displayed significant anti-arthritic effects [30]. There are a few recent studies on polysaccharides from *A. annua* [26,27]. A water-soluble polysaccharide extract was reported to display significant anti-tumor activity, which inhibited HepG2 cell growth by inducing caspase-dependent mitochondrial apoptosis and inhibited NF-κB p65 [27]. The homogeneous polysaccharides from *A. annua* displayed significant anticomplement activities [26]. To the best of our knowledge, there is limited study involving polysaccharides from *A. annua* in modulating the immune system.

Preliminary studies carried out in the authors’ laboratory strongly indicated that *A. annua* has good potential as a source of immunomodulatory and anticancer polysaccharides [31]. The study involved hot water extraction of crude polysaccharides from several Traditional Chinese Medicinal (TCM) herbs, and evaluation of their biological activities with a view to identify the best herbs for further detailed study. The study [31] indicated that *A. annua* was the herb of choice for the isolation of pure polysaccharides and to study their immunomodulatory potential. This paper describes the aqueous extraction and fractionation (based on molecular weight) of polysaccharides from *A. annua* and the evaluation of the antioxidant and immunomodulatory activities of each fraction. Appropriate modulation of the immune system and reducing oxidative stress by the polysaccharides are key considerations when formulating anticancer treatment protocols. Hence, we have evaluated the immunomodulatory and antioxidant potentials of these polysaccharides. Structural characterization of these polysaccharides has also been carried out to further understand their structure–activity relationship and mechanisms of action.

## 2. Results and Discussion

### 2.1. Fractionation and Purification of Polysaccharides from A. annua

**Figure 1:**
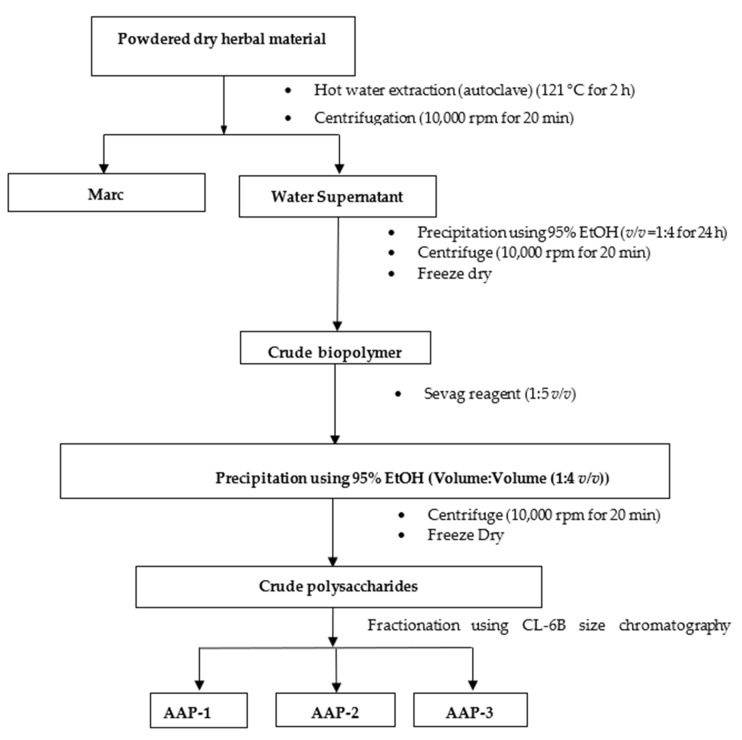
Flow chart for the extraction of polysaccharides from *A*. *annua* [21,32].

**Figure 2:**
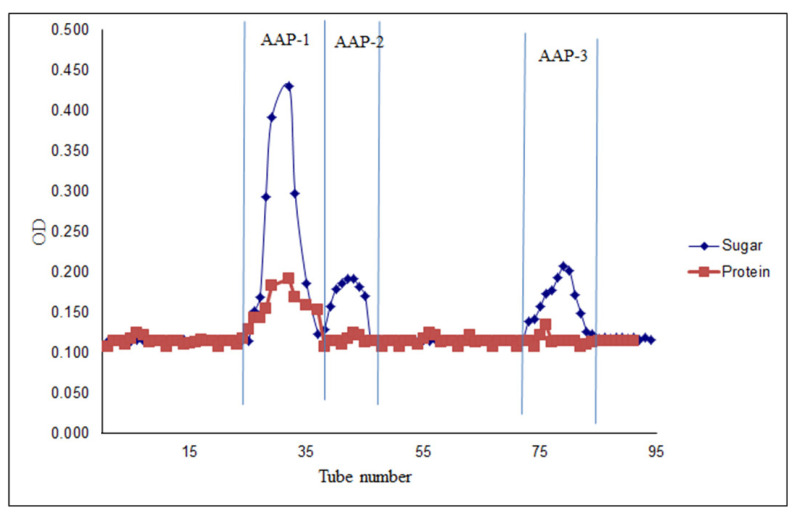
Gel filtration chromatograms of polysaccharide fractions from *A. annua*.

Polysaccharides were extracted from *A. annua* (AAPs) and fractionated by Sepharose CL-6B size-exclusion chromatography. The detailed procedure for the extraction of polysaccharides and their fractionation is shown in Figure 1 [21,32]. Three fractions were selected based on the total sugar profile of the fractions (Figure 2) obtained by the phenol-sulfuric acid method. These crude polysaccharide fractions were designated as AAP-1, AAP-2, and AAP-3. The profile presented in Figure 2 also gives the protein profile of the fractions (Red trace). AAP-1 displayed the highest sugar content and the other two fractions had relatively low sugar content.

**Figure 3:**
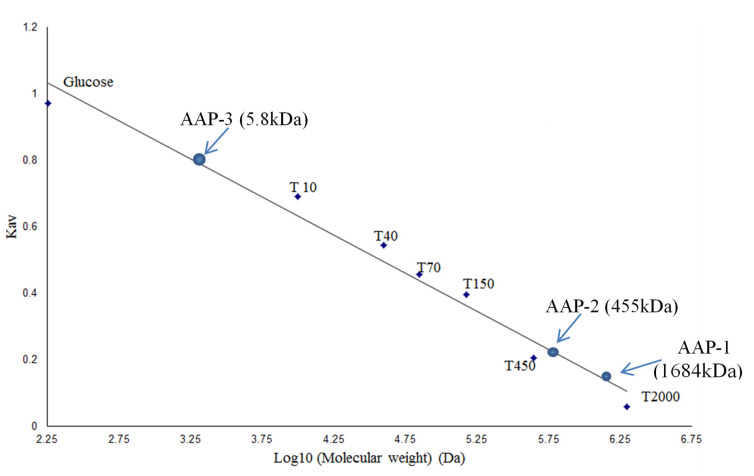
Calibration curve for the determination of molecular weights of polysaccharides from *A. annua* based on the elution volume and the molecular mass of standard dextran series of T2000 (2000 kDa), T450 (450 kDa), T150 (150 kDa), T70 (70 kDa), T40 (40 kDa), T10 (10 kDa) and glucose (180 Da) (Note: Kav = (V_e_ − V_o_)/(V_t_ − V_o_), V_o_ is void volume, V_t_ is total volume and V_e_ is elution volume).

The results for carbohydrate and protein contents in each of the fractions are presented in Table 1. Highest carbohydrate content was observed in AAP-1 (51.8%), followed by AAP-3 (26.3%) and least carbohydrate content was observed in AAP-2 (21.9%). Using the calibration curve obtained from the analysis of dextran molecular weight standards (Figure 3), the average molecular masses of the three fractions, AAP-1, AAP-2 and AAP-3 were determined. The molecular mass of AAP-1 was relatively large, with an estimated average of 1684 kDa, followed by AAP-2 and AAP-3 at 455 and 5.8 kDa, respectively (Figure 3). Table 1 shows the total sugar, protein and monosaccharide content of the three fractions. AAP-1 consists mainly of arabinose (33.35%), glucose (8.69%) and galactose (30.92%), whereas AAP-2 is made up of arabinose (36.02%), glucose (22.16%), mannose (18.17%) and galactose (16.07%) and AAP-3 is mannose (53.08%) and glucose (46.92%). It should be noted that the reduction of fructose yields mannitol and glucitol during GC sample preparation [30,32,33], so it is possible that AAP-3 might be 100% fructose. This aspect is discussed along with the FTIR results. AAP-1 and AAP-2 consist primarily of glucose, galactose, mannose and arabinose.

### 2.2. FTIR Spectroscopic Characterisation of Active Polysaccharides

**Figure 4:**
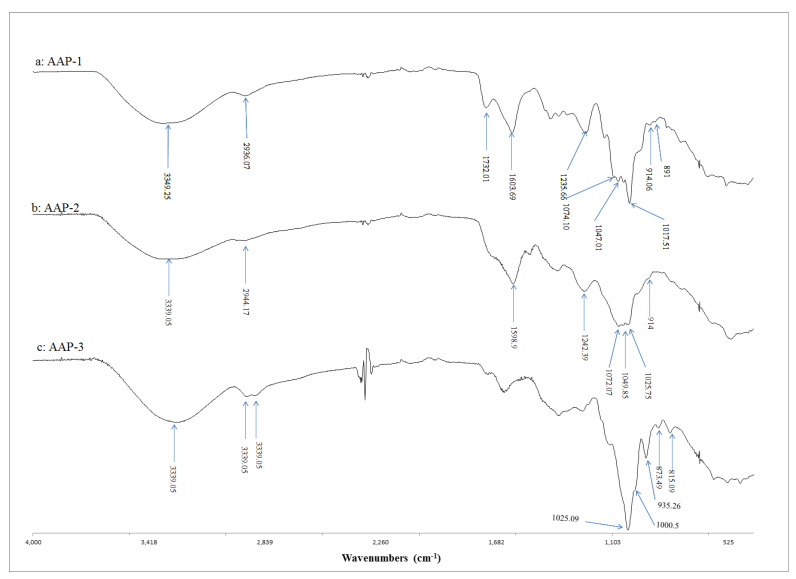
FTIR spectra of the three fractions from *A*. *annua.* (**a**): AAP-1, (**b**): AAP-2 and (**c**): AAP-3.

Figure 4 presents the FTIR spectra of *A. annua* polysaccharides (AAP-1, AAP-2 and AAP-3). The spectrum of AAP-1 showed peaks corresponding to β-glycosidic linkage (914–891 cm^−1^) (Figure 4a) [21,34,35]. The spectrum of AAP-1 also showed three strong absorption bands at 1017.51, 1047.01 and 1074.10 cm^−1^ (corresponding to C-O stretching vibrations related to glycosidic linkage), indicating the presence of pyranose sugar in AAP-1 [21,34,35]. The rest of the vibrational bands conform to a polysaccharide structure. The broad band centered at 3394.25 cm^−1^ corresponds to the hydroxyl stretching vibrations of the polysaccharide and the peak at 2934.07 cm^−1^ belongs to C-H stretching vibrations [21,35]. These observations lead to the conclusion that AAP-1 contains pyranose sugars with β-glycosidic linkages.

The spectrum of AAP-2 (Figure 4b) has a peak at 914 cm^−1^, indicating the presence of β-glycosidic linkage [21,35]. The three strong absorption peaks in the range 1025–1072 cm^−1^ (corresponding to C-O stretching vibrations related to glycosidic linkage) indicate the presence of pyranose sugar [21,35]. The broad band centered at 3339 cm^−1^ corresponds to hydroxyl stretching vibrations and the peak at 2944 cm^−1^ belongs to C-H stretching vibrations. These observations confirm that AAP-2 contains pyranose sugars with β-glycosidic linkages.

The FTIR spectrum of AAP-3 (Figure 4c) is distinctly different compared to that of AAP-1 and AAP-2. In particular, the region between 815–1025 cm^−1^ shows different structural features for AAP-3. The peaks at 873 and 815 cm^−1^ indicate the presence of α- as well as β-glycosidic linkages [21,35]. Two strong absorption peaks in the range of 1000–1100 cm^−1^ (corresponding to C-O stretching vibrations related to glycosidic linkage) indicate the presence of furanose sugars in AAP-3 [21,35]. It is important to note the absence of pyranose sugars in AAP-3 (as there are only two strong absorption bands in the range of 1000–1100 cm^−1^). These spectral features together with the GC findings strongly indicate that AAP-3 is a fructan (Figure 4c) [21,35]. The broad band centered at 3280.6 cm^−1^ corresponds to hydroxyl stretching vibrations of the polysaccharide and the peaks at 2929 and 2889 cm^−1^ belong to the C-H stretching vibrations. These observations indicate that AAP-3 mainly contains furanose sugars with α- and β-glycosidic linkages. These findings together with the results presented in the author’s preliminary paper [32] for LCP-2 indicates that AAP-3 may be a β-fructofuranan. It will be interesting to study the detailed structure of AAP-3 using NMR spectroscopy. This was not possible in this study as the size exclusion separation gave a very small quantity of this fraction.

### 2.3. Antioxidant Activities of AAPs

**Figure 5:**
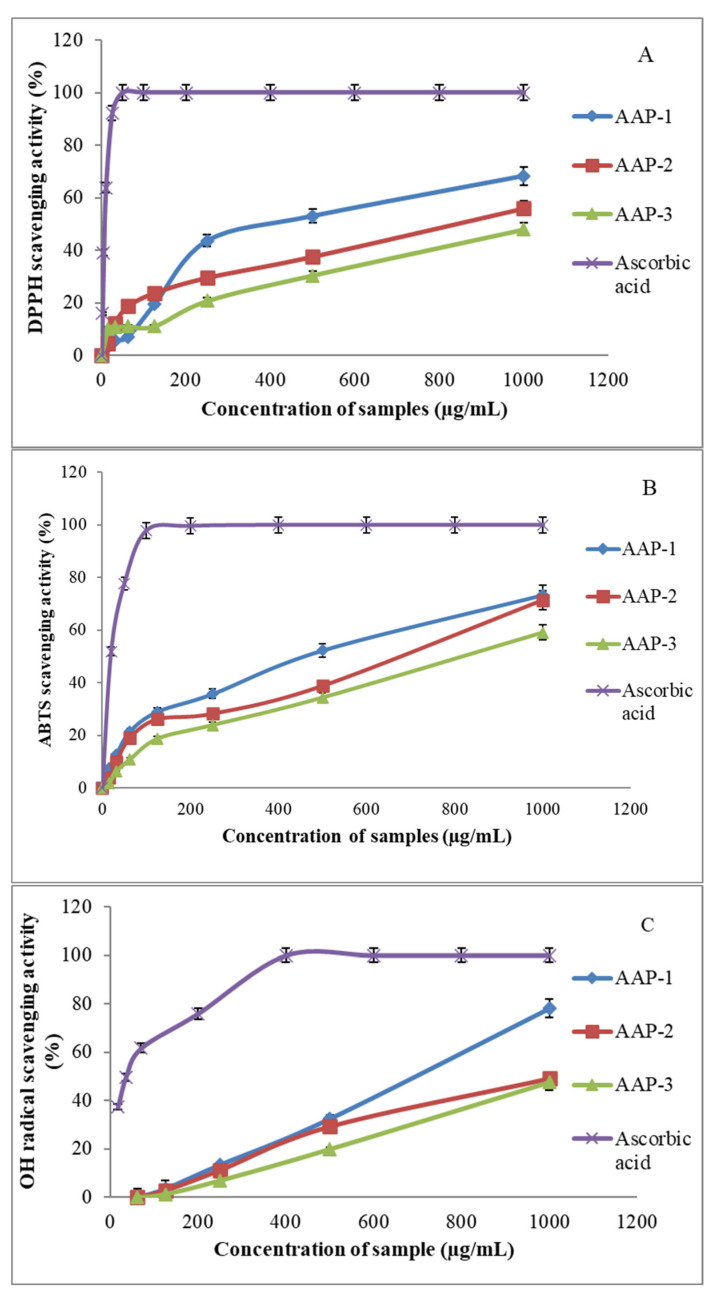
Antioxidant activities of polysaccharide fractions of *A*. *annua*. (**A**) DPPH free radical scavenging activity of the AAPs. (**B**) ABTS free radical scavenging activity of the AAPs. (**C**) Hydroxyl radical scavenging activity of the AAPs. Results are the mean ± SD of three separate experiments, and all the results were compared with the standard (ascorbic acid).

The results of the free radical scavenging capacity of these polysaccharide fractions are presented in Figure 5. The three fractions displayed significant DPPH^●^ and ABTS^●+^ radical scavenging capacities (Figure 5A,B). The most active fraction against DPPH^●^ was AAP-1 (68%) at a concentration of 1000 μg/mL, and the least active was AAP-3 (47%) at 1000 μg/mL. The trend against ABTS^●+^ radical was similar to AAP-1, displaying 73% activity, whereas AAP-3 was 59% at 1000 μg/mL concentration. The EC50 value for DPPH radical scavenging activity of AAP-1 was about 426 µg/mL, followed by AAP-2 (845 µg/mL) and AAP-3 (1392 µg/mL). The EC50 value for ABTS radical scavenging activity of AAP-1 was about 392 µg/mL, followed by AAP-2 (553 µg/mL) and AAP-3 (798 µg/mL). The hydroxyl (OH^●^) radical scavenging abilities of the polysaccharides are presented in Figure 5C. AAP-1 showed extremely high OH^●^ scavenging activity (more than 70%), followed by AAP-2, whereas AAP-3 was the least active at 1000 μg/mL concentration. The EC50 value for the hydroxyl (OH^●^) radical scavenging ability of AAP-1 was about 630 µg/mL. However, the antioxidant activities of the polysaccharides are lower than the activity of ascorbic acid (Figure 5).

Literature reports indicate that various factors can influence the antioxidant capacities of botanical polysaccharides [34,36,37]. Major factors that contribute to enhanced activity are (i) higher average molecular weight (more than 90 kDa) for antioxidant activity [37], (ii) presence of β-glycosidic linkages [32,37], (iii) presence of large quantities of glucose, galactose, rhamnose and arabinose within the polysaccharide structure [34,36,37], and (iv) presence of protein or peptide conjugation to the polysaccharide chain increases the radical scavenging ability [21,37].

The results presented in this research are in good agreement with those reported in the literature [34,36,37]. For example, AAP-1 with highly significant antioxidant activity has (i) high average molecular weight (Figure 3), (ii) contains β-glycosidic linkages (Figure 4a), (iii) has protein conjugation (29% protein content) and (iv) contains glucose, galactose, rhamnose and arabinose (Table 1).

### 2.4. Immunomodulatory Effects of Polysaccharides from A. annua

**Figure 6:**
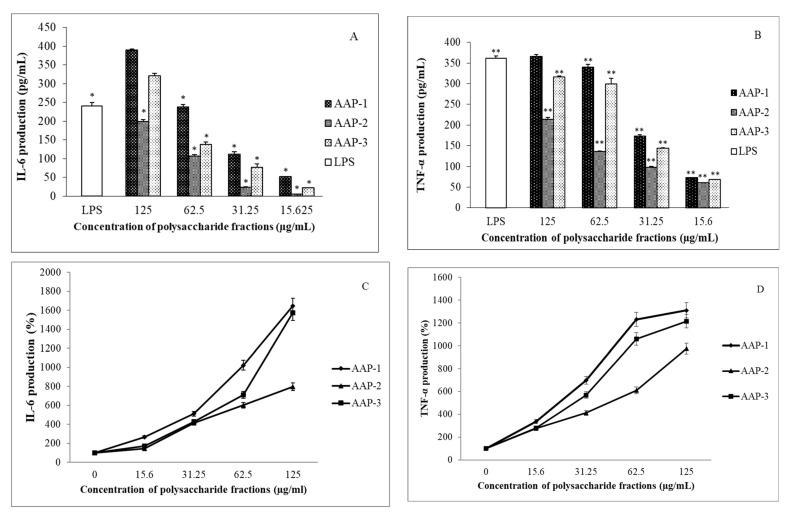
Effects of *A*. *annua* polysaccharides on murine RAW 264.7 macrophages. (**A**,**C**): represent interleukin 6 (IL-6) production and (**B**,**D**): represent tumor necrosis factor-α (TNF-α) production. * Statistical difference for the positive control (LPS treated group) and the samples was significant, n = 3, *p* < 0.05. ** Statistical difference for the positive control (LPS treated group) and the samples was significant, n = 3, *p* < 0.03.

Immunomodulatory activities of the three isolated polysaccharide fractions were determined by the treatment of RAW 264.7 macrophages with AAPs, and the results for the production of TNF-α and IL-6 are presented in Figure 6.

The results from Figure 6 show that the AAPs have highly significant immunomodulatory activity as indicated by increasing IL-6 and TNF-α production in a dose-dependent manner (Figure 6). As can be seen from Figure 6C,D, the immunostimulatory activities of AAP-1, AAP-2 and AAP-3 increase sharply when the polysaccharide concentration is greater than 15 µg/mL. Excellent immunostimulatory activities are observed for AAP-1 at 125 µg/mL as indicated by: (i) an over 16-fold increase in the production of IL-6 compared to the control (untreated macrophages) (Figure 6C) and (ii) a nearly 13-fold increase in the production of TNF-α (Figure 6D). Immunostimulatory activities of AAP-2 at 125 µg/mL had: (i) a nearly 8-fold increase in the production of IL-6 compared to the control (untreated macrophages) (Figure 6C) and (ii) a nearly 10-fold increase in the production of TNF-α (Figure 6D). Highly significant immunostimulatory activities were also observed for AAP-3 at 125 µg/mL as indicated by: (i) an over 15-fold increase in the production of IL-6 compared to the control (untreated macrophages) (Figure 6C) and (ii) a more than 12-fold increase in the production of TNF-α (Figure 6D). These observations are very significant and demonstrate that AAP-1, AAP-2 and AAP-3 are highly suitable candidates for stimulating the immune system.

As discussed before, appropriate modulation of the immune system and reducing oxidative stress are the important properties to be considered when designing anticancer therapeutics/formulations [21,22,25,32]. *A*. *annua* polysaccharides reported in this research displayed highly significant immunomodulatory and antioxidant activities. It is therefore proposed that AAPs are potential candidates for the development of effective anticancer formulations. Further research in this direction is underway in the authors’ laboratory.

**Figure 7:**
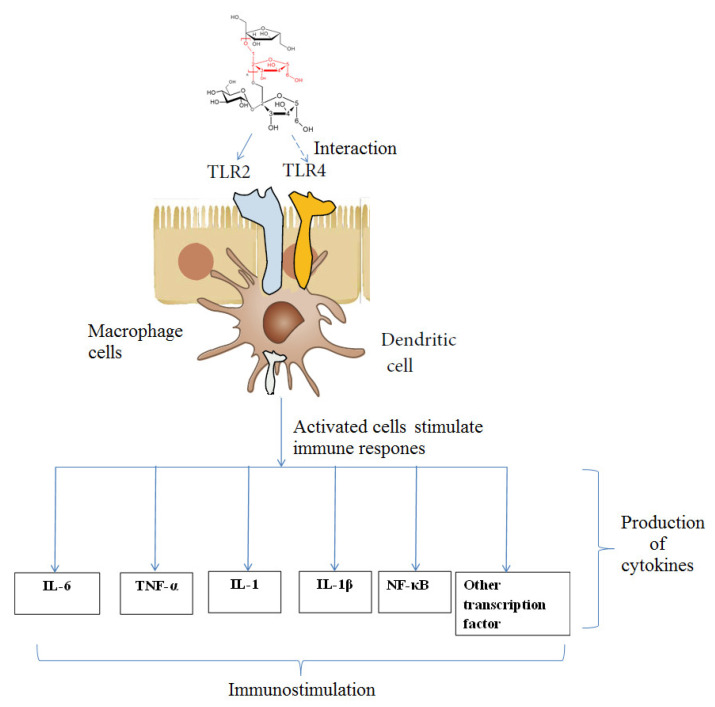
Schematic representation of mechanism of immunostimulatory activity induced by inulin-type fructans (β-D-(2→1)-fructan) of longer chain lengths (Note: Fructans with shorter chains leads to decreased production of cytokines) [40,41].

Information from the literature indicates that toll-like receptors (TLR) can recognize and bind with various types of polysaccharides such as protein-polysaccharide complexes, inulins and glucans and activate macrophages to promote cytokine secretion (Figure 7) [38,39]. For instance, high molecular weight polysaccharide–protein complex isolated from *Lentinus edodes* displayed significant immunomodulatory activities [38].

These observations are consistent with literature findings that plant polysaccharides can display immunomodulatory activities [32,39,40,41].

The structure of AAP-3 (Section 2.2) has been identified as a β-(2→1)-fructan with an average molecular mass of 5.8 kDa. Fructans of this size are known to possess significant immunostimulatory activities [32,40,41]. β-(2→1)-linked fructans with long chain lengths (consisting of 11–60 fructose units) can directly interact with dendritic cells (DCs) (Figure 7) [39]. The toll-like receptors (TLRs) present on DCs and macrophages recognize β-(2→1)-linked fructans (inulin-type fructans) and can activate TLR-2 to stimulate immune response and produce cytokines (such as IL-6, IL-1 and TNF-α) [32,39,40,41]. Fructans mainly activate TLR-2, but also TLR-4 and other TLRs to a lesser extent [40,41]. It has also been recognized that β-fructan chain length is an important factor in this mechanism of action (Figure 7) with long chain lengths favoring better immunostimulatory activity [32,40,41].

Two β-(2→1)-linked fructans (inulin type immunomodulatory fructans) have been discovered in the authors’ laboratory: (i) LCP-2 with an average molecular mass of 5.3 kDa published earlier [32] and (ii) AAP-3 with an average molecular mass of 5.8 kDa. Consistent with the literature [35,36], AAP-3 with a larger molecular mass showed significantly higher immunostimulatory activity than LCP-2 [32].

Abundant literature in this area indicates that immunomodulatory activity plays an important role in cancer treatment [19,20,21,22,23,24,25,26,27,28,29,30,31]. Hence, *A. annua* polysaccharides isolated in this study are potential anticancer agents and useful for the development of anticancer formulations. 

### 2.5. Cell Viability

**Figure 8:**
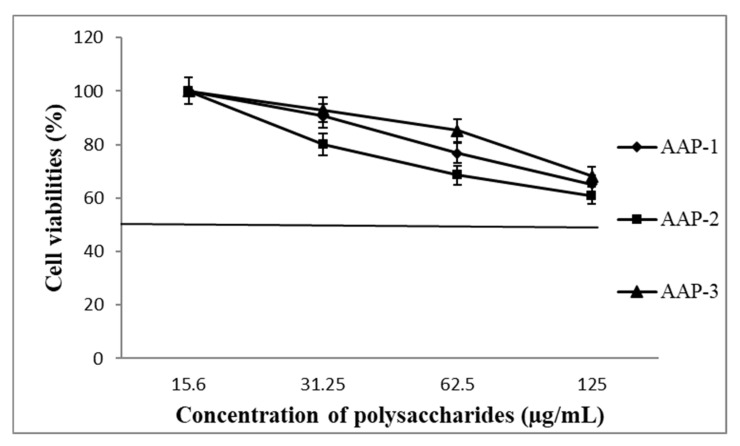
Cell viabilities of isolated polysaccharide fractions from *A. annua* at different concentrations.

The effect of AAP-1, AAP-2 and AAP-3 on the viability of mouse macrophage cells is given in Figure 8. The result that the polysaccharides from *A. annua* show significant cell viabilities even at the highest concentration (125 µg/mL) used in this study indicate that they have low toxicity. These results are consistent with literature reports that plant polysaccharides are essentially non-toxic [21,25,31,32].

## 3. Materials and Methods

### 3.1. Material

*Artemisia annua* (Qing Hao, aerial part) was purchased from Herbal Life Chinese Herbal Medicine shop, Sydney, Australia. The herbs traded in Australia have approvals from both Australian and Chinese governments. Specifications from the supplier indicate that the plant was harvested at the optimum time for medicinal efficacy.

### 3.2. Chemicals

The DPPH^●^, ABTS^●+^, 1,10-phenanthroline, H_2_O_2_, dimethyl sulfoxide (DMSO), 95% ethanol, ascorbic acid, trypan blue 0.4%, and lipopolysaccharide (LPS) were purchased from Sigma (Rowville, Australia) and Lomb Scientific Pty Ltd. (Sydney, Australia). The foetal bovine serum (FBS), antibiotics, and Dulbecco’s modified Eagle’s medium (DMEM) with gluMax were purchased from BD Bioscience (USA). The tumor necrosis factor-α (TNF-α) and interleukin (IL-6) (mouse)—ELISA standards and antibodies were also purchased from BD Bioscience.

### 3.3. Extraction and Fractionation of Polysaccharides from A. annua

To extract the water-soluble compounds, 500 g of dried *A. annua* was powdered then autoclaved (2000 mL, 121 °C, 2 h). Details of the procedure are similar to that published previously and is described in Figure 1 [21,31,32]. These fractions were collected and concentrated by freeze drying, then stored at −20 °C for further studies.

### 3.4. Determination of Molecular Weights of Polysaccharide Fractions

Estimation of molecular weights of the purified polysaccharide fractions was done on the basis of the elution volume and molecular weight using a standard dextran series that included T2000 (2000 kDa), T450 (450 kDa), T150 (150 kDa), T70 (70 kDa), T40 (40 kDa), T10 (10 kDa) and glucose at a concentration of 10 mg/mL each for calibrating the Sepharose CL-6B column [21,31,32]. Regression of the standard curve gave a linear equation (with R^2^ = 0.9882) represented by:

y = −0.2328x + 1.523(1)

which was used to estimate the average molecular weight of the polysaccharides. 

### 3.5. Analysis of Monosaccharides 

The total sugar content was measured using the phenol–sulfuric acid method [21,32]. Glucose was used to produce a standard curve for determining the sugar content. Regression of the standard curve gave a linear equation (R^2^ = 0.9964) represented by:

y = 0.0018x + 0.0374(2)

The total protein content was measured using a modified Lowry’s method, where BSA was used to prepare the standards [21,32] for constructing the standard curve for determining the bound protein. Regression of the standard curve gave a linear equation (R^2^ = 0.9923) represented by:

y = 0.0017x − 0.0212(3)

The mono-sugar content was determined by gas chromatography (Hewlett Packard 7890B) with FID detection [21,32]. The approach followed to prepare the samples for GC analysis was based on the procedure published previously [21]. Mannose, glucose, galactose, xylose, fucose, rhamnose, arabinose and ribose were used as mono-sugar standards.

### 3.6. Bioactivity Tests

#### 3.6.1. DPPH^●^ Scavenging Assay

The Blois method was employed to determine the DPPH^●^ scavenging ability of the polysaccharides and is similar to that described in previous publications [32,42,43].

Free radical scavenging activities of AAPs was evaluated using the equation:

(4) $${\mathrm{DPPH}}^{●}\;\mathrm{scavenging}\;\mathrm{activity}\;(\%)=\frac{OD_{control}-OD_{Sample}}{OD_{control}}\;\times \;100\%$$

where OD of the control is the absorbance of DPPH solution without sample and OD of sample is the test sample (DPPH solution plus test sample or positive control).

#### 3.6.2. ABTS^●+^ Radical Scavenging Assay

ABTS^●+^ scavenging ability of the polysaccharides was determined using a published method and is similar to that described in previous publications [32,43].

Free radical scavenging activity of AAPs was evaluated using the equation:

(5) $${\mathrm{ABTS}}^{●+}\;\mathrm{scavenging}\;\mathrm{activity}\;(\%)=\frac{OD_{control}-OD_{Sample}}{OD_{control}}\;\times \;100\%$$

where OD of the control is the absorbance of ABTS solution without sample and OD of sample is the test sample (ABTS solution plus test sample or positive control).

#### 3.6.3. OH^●^ Radical Scavenging Assay

The OH^●^ scavenging assay was a slightly modified method described by de Avellar et al., (2004) [44]. The procedure is similar to that presented in previous publications [32]. Ascorbic acid (Vc) at a concentration range of 17–1000 μg/mL was the positive control. The OH^•^ scavenging ability of AAPs was determined using the equation:

(6) $${\mathrm{OH}}^{•}\;\mathrm{scavenging}\;\mathrm{activity}\;(\%)=\frac{OD_{Sample}-OD_{neg\_control}}{OD_{blank}-OD_{neg\_control}}\;\times \;100\%$$

where the negative control is the reaction mixture without sample and without ascorbic acid. The blank is the reaction mixture without sample, ascorbic acid and H_2_O_2_.

#### 3.6.4. Immunomodulatory Activity Assays

Mouse macrophages (RAW 264.7) were first added to DMEM (culture medium containing 1% antibiotic and 5%FBS) and incubated for 4 days at 37 °C in 5% CO_2_. The cells were then diluted with the medium to achieve a density of 2 × 10^5^ cells/mL. The procedure is based on the published literature [32].

##### IL-6 Production

ELISA kit (IL-6, BD Biosciences, San Jose, CA, USA) was used to measure the concentration of IL-6 following the procedure in the manufacturer’s manual [31,32,45,46]. All experiments were conducted in triplicate. Standard IL-6 (mouse) was used to produce a calibration curve that gave the linear equation (R^2^ = 0.992):

y = 0.0019x + 0.0248(7)

which was then used to determine the concentration of IL-6 produced by the polysaccharide extract.

##### TNF-α Production

ELISA kit (TNF-α, BD Biosciences, San Jose, CA, USA) was used to measure the concentration of TNF-α following the method provided in the manufacturer’s manual and as previously described [31,32,45,46]. Triplicate measurements were made. 

Standard TNF-α (mouse) was used to produce the calibration curve that gave the linear equation (R^2^ = 0.9867):

y = 0.0015x + 0.067(8)

which was used to determine the concentration of TNF-α produced by the polysaccharide extract.

#### 3.6.5. Determination of Cell Viability by MTT Assay

Viability of macrophage cells (RAW 264.7) was measured using the MTT assay as previously described [31,32]. The absorbance was measured at 595 nm and the fraction of live cells was determined using the equation:

(9) $$Cell\;viability\;\left(\%\right)=\frac{OD\;of\;sample}{OD\;of\;pos\;control}\times 100\%$$

The positive control was mouse macrophages treated by only DMEM medium (without LPS and sample).

### 3.7. Fourier Transform Infrared (FTIR) Spectroscopy

A TENSOR II FTIR spectrometer (BRUKER) was used for structural characterization of the AAPs at room temperature (25 °C) [32,34]. All spectra were recorded between 4000–450 cm^−1^.

### 3.8. Statistical Analysis

Data is expressed as mean ± standard deviation (SD) values. The group mean was compared using a one-way analysis of variance (ANOVA) and Duncan’s multiple range tests. The statistical difference was considered significant if *p* < 0.05. All statistical analyses were performed using OriginPro 8.5 and Excel 2016.

## 4. Conclusions

Three polysaccharide fractions were successfully isolated from the aqueous extract of *A. annua* (AAP-1, AAP-2 and AAP-3). AAPs isolated from *A. annua* displayed highly significant immunostimulatory capacities and antioxidant activities. In particular, AAP-1 and AAP-3 have displayed very high immunostimulatory activities and low toxicity, demonstrating that they have high potential as natural immune-enhancing agents. 

FTIR results indicate that AAP-1 and AAP-2 are pyranose-containing polysaccharides with β-linkages. GC and FTIR results lead to the conclusion that AAP-3 is a β-fructofuranoside. It is pertinent to further compare the biological activity results and structural features of LCP-2 [32] with those of AAP-3. Both polysaccharides gave very similar mono-sugar ratios, similar FTIR spectral features and very comparable biological activities. These results strongly indicate that AAP-3 is a β-fructan. 

## Figures and Tables

**Table 1:** Sugar composition of polysaccharide fractions isolated from *A. annua*.

	AAP-1	AAP-2	AAP-3 *
Protein (%)	29.57	10.95	9.32
Carbohydrate (%)	70.43	89.05	90.68
Monosaccharide (% ratio)			
Rhamnose (%)	9.67		
Ribose (%)			
Fucose (%)			
Arabinose (%)	33.35	36.02	
Xylose (%)	7.67		
Mannose (%)	1.44	18.17	53.09
Galactose (%)	30.92	16.07	
Glucose (%)	8.69	22.16	46.92
Unknown (%)	8.26	7.58	

* It should be noted that the reduction of fructose yields mannitol and glucitol during GC sample preparation. It is therefore likely that AAP-3 is 100% fructose.

## Data Availability

Not applicable.

## Sample Availability

Samples of the compounds are not available from the authors.
